# Coral restoration and adaptation in Australia: The first five years

**DOI:** 10.1371/journal.pone.0273325

**Published:** 2022-11-30

**Authors:** Ian M. McLeod, Margaux Y. Hein, Russ Babcock, Line Bay, David G. Bourne, Nathan Cook, Christopher Doropoulos, Mark Gibbs, Peter Harrison, Stewart Lockie, Madeleine J. H. van Oppen, Neil Mattocks, Cathie A. Page, Carly J. Randall, Adam Smith, Hillary A. Smith, David J. Suggett, Bruce Taylor, Karen J. Vella, David Wachenfeld, Lisa Boström-Einarsson

**Affiliations:** 1 TropWATER (Centre for Tropical Water and Aquatic Ecosystem Research), James Cook University, Townsville, Queensland, Australia; 2 Australian Institute of Marine Science, Townsville, Queensland, Australia; 3 MER Research and Consulting, The Office, Monaco, Monaco; 4 CSIRO Oceans & Atmosphere, St Lucia, Queensland, Australia; 5 College of Science and Engineering, James Cook University, Townsville, Australia; 6 Reef Ecologic, Townsville, Queensland, Australia; 7 Marine Ecology Research Centre, Faculty of Science and Engineering, Southern Cross University, Lismore, New South Wales, Australia; 8 The Cairns Institute, James Cook University, Cairns, Queensland, Australia; 9 School of BioSciences, University of Melbourne, Parkville, Victoria, Australia; 10 Great Barrier Reef Marine Park Authority, Townsville, Queensland, Australia; 11 School of Biological, Earth and Environmental Sciences, University of New South Wales, Randwick, New South Wales, Australia; 12 Climate Change Cluster, University of Technology Sydney, Ultimo, New South Wales, Australia; 13 Land & Water, Commonwealth Scientific and Industrial Research Organisation, Dutton Park, Queensland, Australia; 14 School of Architecture and Built Environment, Queensland University of Technology, Brisbane, Australia; 15 Lancaster Environment Centre, Lancaster University, Bailrigg, Lancaster, United Kingdom; Newcastle University, UNITED KINGDOM

## Abstract

While coral reefs in Australia have historically been a showcase of conventional management informed by research, recent declines in coral cover have triggered efforts to innovate and integrate intervention and restoration actions into management frameworks. Here we outline the multi-faceted intervention approaches that have developed in Australia since 2017, from newly implemented in-water programs, research to enhance coral resilience and investigations into socio-economic perspectives on restoration goals. We describe in-water projects using coral gardening, substrate stabilisation, coral repositioning, macro-algae removal, and larval-based restoration techniques. Three areas of research focus are also presented to illustrate the breadth of Australian research on coral restoration, (1) the transdisciplinary Reef Restoration and Adaptation Program (RRAP), one of the world’s largest research and development programs focused on coral reefs, (2) interventions to enhance coral performance under climate change, and (3) research into socio-cultural perspectives. Together, these projects and the recent research focus reflect an increasing urgency for action to confront the coral reef crisis, develop new and additional tools to manage coral reefs, and the consequent increase in funding opportunities and management appetite for implementation. The rapid progress in trialling and deploying coral restoration in Australia builds on decades of overseas experience, and advances in research and development are showing positive signs that coral restoration can be a valuable tool to improve resilience at local scales (i.e., high early survival rates across a variety of methods and coral species, strong community engagement with local stakeholders). RRAP is focused on creating interventions to help coral reefs at multiple scales, from micro scales (i.e., interventions targeting small areas within a specific reef site) to large scales (i.e., interventions targeting core ecosystem function and social-economic values at multiple select sites across the Great Barrier Reef) to resist, adapt to and recover from the impacts of climate change. None of these interventions aim to single-handedly restore the entirety of the Great Barrier Reef, nor do they negate the importance of urgent climate change mitigation action.

## Introduction

Historically, management of coral reefs in Australia has focused on reducing local and regional stressors. These include, for example, overfishing (mainly through zoning and enforcement), mining for oil and gas exploration, coastal development (through planning, permitting and mitigation), and poor water quality (through land management and improved technology). Apart from targeted crown-of-thorns starfish (COTs) control, which has since 2013 become an important part of reef management on the Great Barrier Reef (GBR) [1], active intervention, and in particular, coral reef restoration has not been part of Australian coral reef management strategies. However, following back-to-back coral bleaching events in 2016–17 and the increase in frequency and intensity of other localised threats (e.g., tropical cyclones and COTs outbreaks [2–5]), coral reef restoration is increasingly considered as a potential tool to accelerate recovery and improve resilience of the GBR [6–8]. The appetite for active intervention is supported by evidence that even under low-emission scenarios, the future of coral reefs is uncertain [9]. Management of the GBR is therefore shifting to consider new interventions to be developed and tested now, so that safe and effective interventions can be implemented alongside conventional management efforts and stronger action to curb global warming (e.g., [7, 10–13]).

Despite coral reef restoration feasibility assessments in the early 1980s [14], research and uptake of coral reef restoration in Australia has remained largely sporadic. Early trials included feasibility assessments of coral transplantation [15], collecting wild coral spawn and raising larvae in small floating larval nursery ponds to enhance settlement processes [16], and research into the efficacy of coral fragment attachment techniques [17]. Transplantation was also used to move colonies of *Porites spp*., away from a harbour development site to avoid physical damage from dredging activities [18].

**Fig 1:**
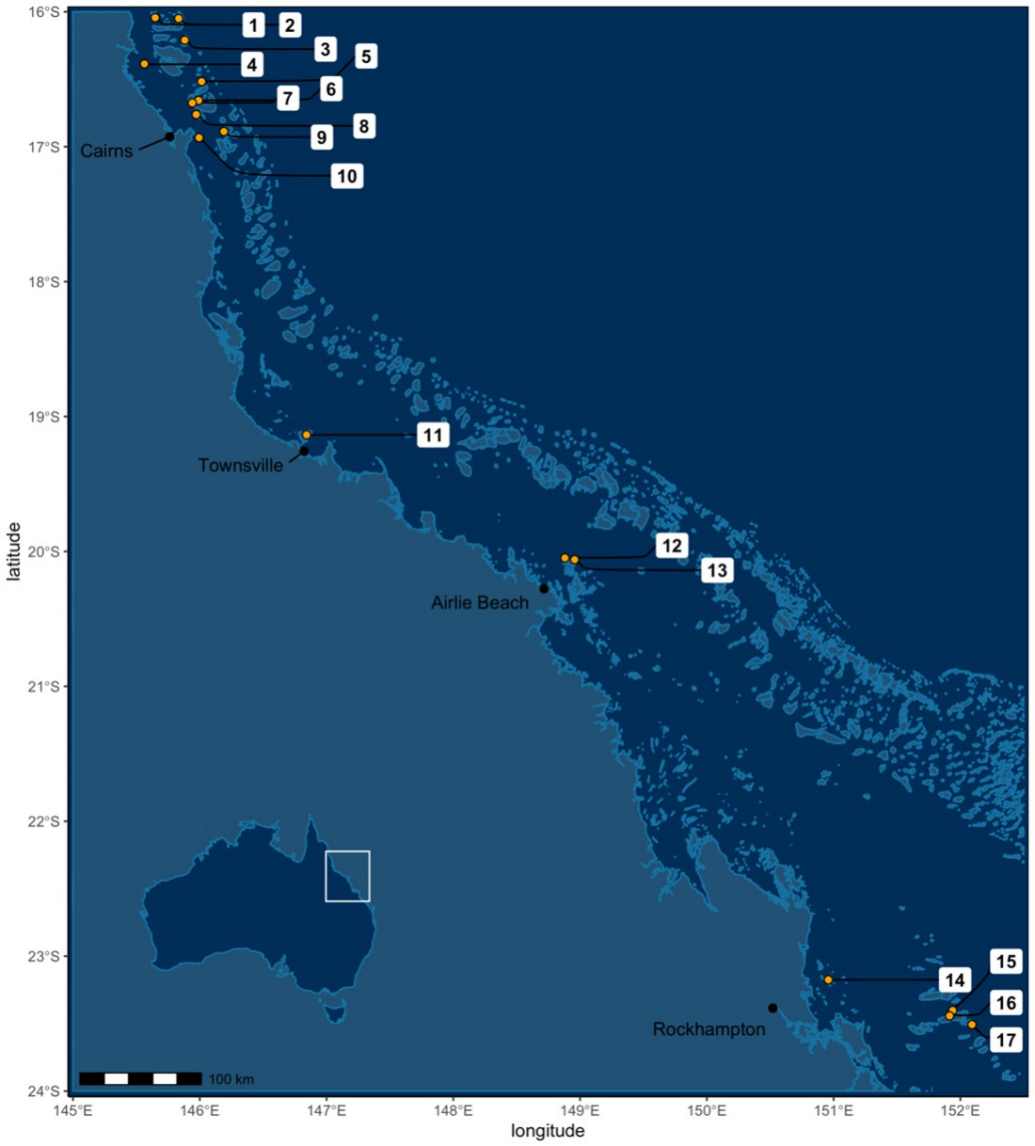
The locations of recent restoration projects on the Great Barrier Reef. 1) Mackay Reef; 2) Agincourt Reef 3; 3) Opal Reef; 4) Low Isles; 5) Hastings Reef; 6) Vlasoff Cay; 7) Upolu Reef; 8) Green Island; 9) Moore Reef (2 projects); 10) Fitzroy Island; 11) Magnetic Island; 12) Blue Pearl Bay; 13); Manta Ray Bay (2 projects); 14) Keppel Islands 15) offshore Heron Island; 16) Heron Island; 17) One Tree Island.

In other regions of the world, research, and implementation of coral restoration approaches have advanced greatly in the last 20 years. Coral reef restoration projects have been reported in at least 56 countries, with the majority happening in the Caribbean and South-East Asia [10], providing an excellent basis for Australia to build upon without having to re-invent the methods (e.g., [19–22]). However, extensive research is still necessary to translate and adapt coral reef restoration methods to specific challenges faced by Australian reefs and to accelerate research and development in new techniques to enhance corals tolerance at scale to adapt to a changing climate. To that end, the Australian Commonwealth Government established the Reef Restoration and Adaptation Program (RRAP) in 2018 [23] with the mission to develop interventions and delivery methods focused on assisting the GBR’s potential to recover from major disturbances and adapt to a changing climate. RRAP is now one of the largest coral reef research initiatives globally. Independent from RRAP, there are also approximately 19 current coral restoration projects underway at 17 distinct locations on the GBR (Fig 1, S1 Appendix). All the restoration and adaptation work in Australia involve a range of tourism operators, reef managers, local community groups and Traditional Owners.

With such a breadth of funding and expertise, there is an opportunity for Australia to add to and further the development of global coral reef restoration research. Building on and learning from the extensive existing body of work in other countries, innovations from Australia may strengthen effective implementation of coral reef restoration globally. This paper presents an overview of the multi-faceted Australian coral restoration projects, from newly implemented, community-led in-water programs, to innovative scientific research to enhance coral adaptation.

### Coral gardening

**Fig 2:**
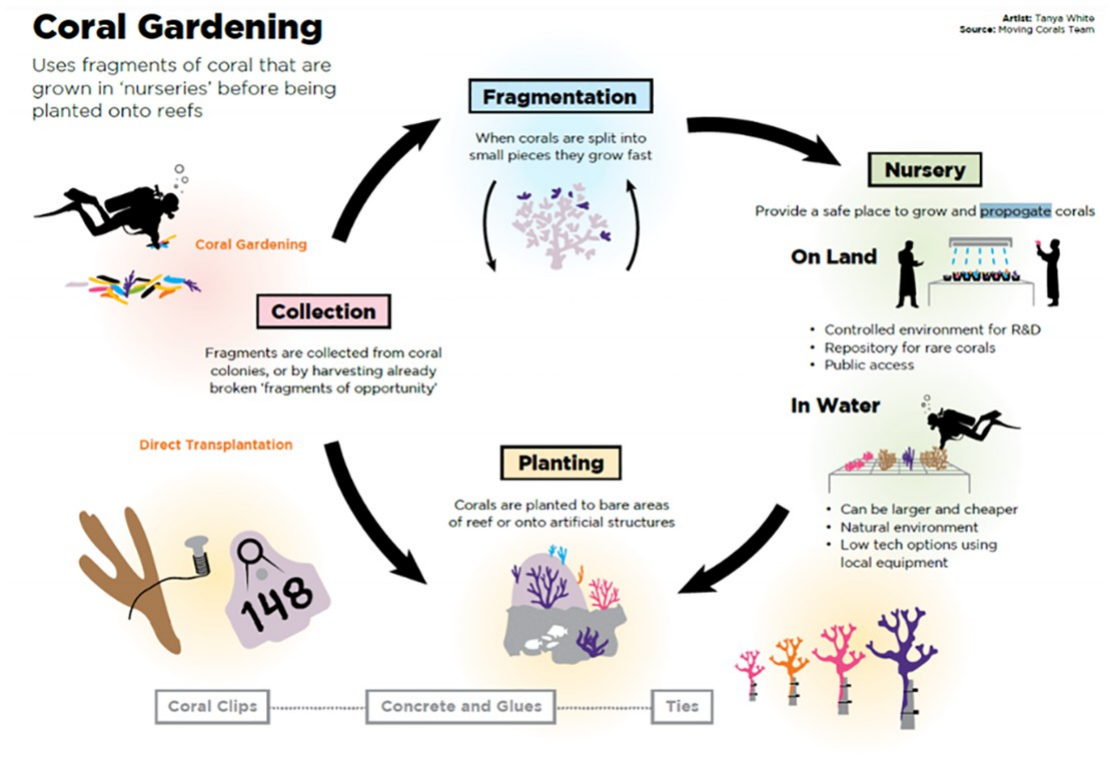
The coral gardening process involves the collection of coral fragments, fragmentation, a nursery phase on land or in water, and an (out)planting phase.

Coral gardening (also referred to as ‘in-water coral propagation’) describes asexual coral propagation methods whereby coral fragments are transplanted back onto a reef after an intermediate nursery phase ([24], Fig 2). Such methods are the most widely used for coral restoration projects around the world [10].

Since 2017, eleven coral gardening projects have been established within the GBR marine park including one project at Fitzroy Island, six projects in the broader Cairns-Port Douglas region, one project in the Townsville region and three projects in the Whitsundays region. The project at Fitzroy Island, near Cairns, was led by the Reef Restoration Foundation (RRF) and was the first in-water coral gardening project to be established on the GBR. The project started in 2017 using the ‘coral tree’ nursery design developed by the Coral Restoration Foundation, in the Florida Keys, USA [25]. The nursery trees were initially populated with fragments from four *Acropora* species collected from donor colonies around the island that had survived the 2016–2017 bleaching [26]. In 2021, the in-water nursery had 20 trees and over 1500 fragments [26]. The fate of individual outplanted corals was not monitored and there was no significant effect of coral planting on live coral cover after 12 months [26].

**Fig 3:**
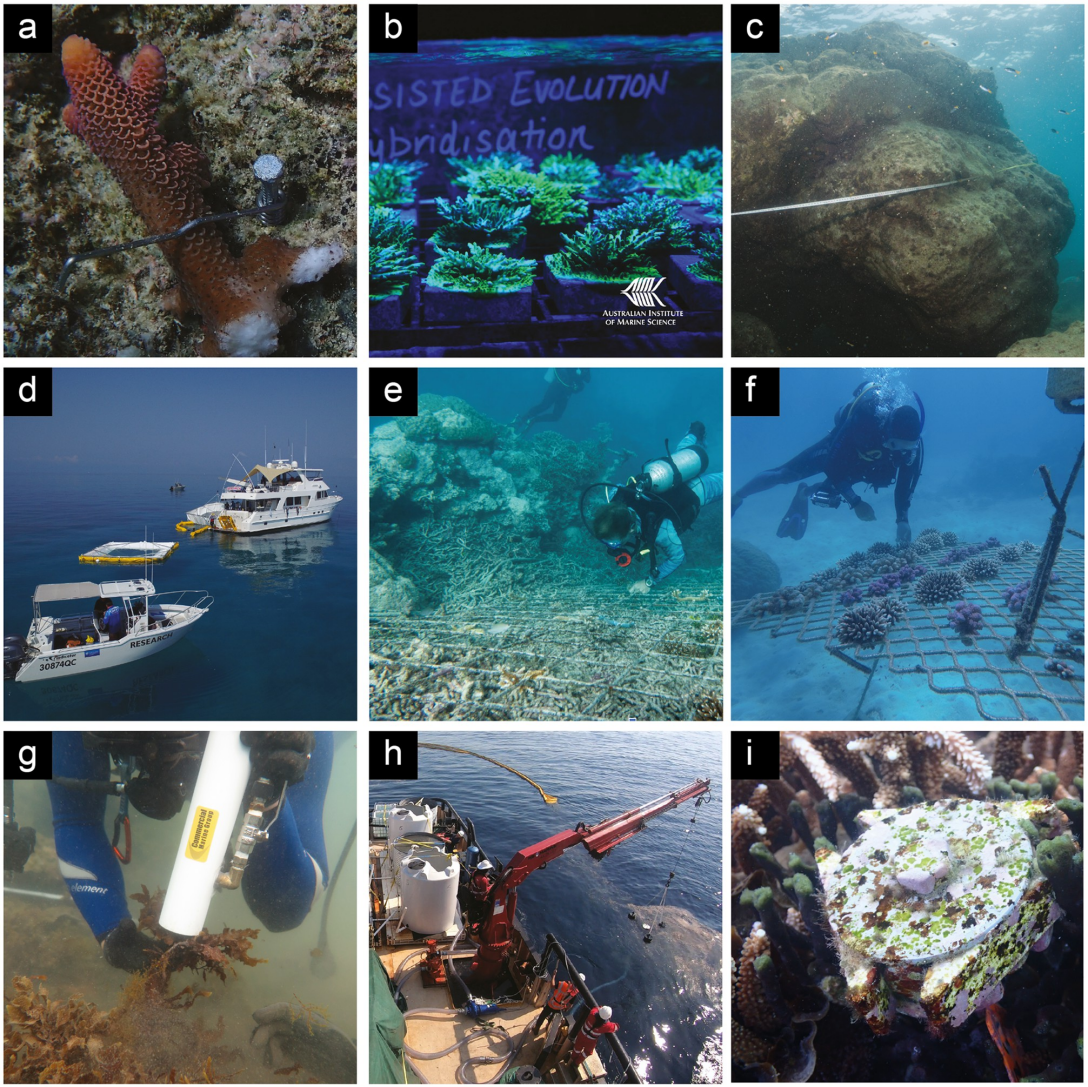
Coral restoration projects and methods currently underway in Australia: a) the CoralClip®, b) assisted evolution research, c) coral repositioning, d) larval-based restoration, e) substrate stabilisation, f) coral gardening, g) ‘supersucker’ for macroalgae removal, h) industrial larval capture, i) coral seeding units. Image credits: J. Edmondson, M. Roman/AIMS, I. McLeod, R. Miller, M. Hein, D. Suggett, N. Mattocks, C. Doropoulos, Cathie Page.

The Coral Nurture Program uses coral gardening techniques at six reefs that are important to the tourism industry in the Cairns-Port Douglas region. This program began in 2018 as a partnership between a local tourism company (Wavelength Reef Cruises) and researchers from the University of Technology, Sydney. Initially, the project established two coral nurseries at Opal Reef (Fig 3f), and developed a new attachment device (Coralclip®, Fig 3a) to outplant both coral fragments and larval settlement devices (small travertine tiles) in a targeted manner [27]. In the first few months of the project, the team outplanted over 3500 coral fragments and established 2500 corals in nurseries [27–29]. In 2019, the program expanded to include four more tourism operators (Ocean Freedom, Passions of Paradise, Quicksilver, and Sailaway) and five more reefs (Hastings Reef, Low Isles, Mackay Reef, Moore Reef, and Upolu Reef). This resulted in >20,000 outplants (and 5,500 corals established in 80 nurseries) by the end of 2020. While still requiring further testing and longer-term monitoring, Coralclip® is showing great promises as a coral reef restoration method in terms of outplanting speed (0.3–1.9 coral fragments per diver per minute), cost (USD$ 0.6–3.0 per coral deployed) and attachment rates (≤15% failure rate of the clip deployment method after 3–7 months, [27]). A key outcome of these activities was the capacity for tourism operators to employ coral restoration activities at the reef-site scale within the framework of stewardship of high-value tourism reef sites. The impacts of COVID-19 included an almost complete shutdown of the tourism industry that focused on the Great Barrier Reef. Through the Australian Government’s $1 billion COVID-19 Relief and Recovery Fund, the Great Barrier Reef Marine Park Authority (GBRMPA) launched the Tourism Industry Activation and Reef Protection Initiative (TIARPI) in 2021 to assist the tourism industry in its recovery. TIARPI enabled tourism operations to repurpose their assets (vessels and staff) to perform contracted services at high-value tourism sites by conducting Eye on the Reef monitoring surveys and undertaking site stewardship activities that included coral restoration.

In November 2018, two coral gardening projects were established in the Whitsundays region (in Blue Pearl Bay and Manta Ray Bay) by environmental consultants (Reef Ecologic) in partnership with a tourism promotion organisation (Tourism Whitsundays) and Daydream Island Resort. At each location, two types of coral nursery were trialled, (1) rope nurseries where coral fragments were fixed between strands of rope and attached to a frame suspended off the seafloor, and (2) table nurseries where coral fragments were attached to cement discs fixed to a tray suspended mid-water. Each nursery held 700–1000 coral fragments, with approximately 50% of fragments surviving until being planted [30]. To date, 3068 coral colonies have been propagated in nurseries and 1312 coral colonies have been outplanted. Monitoring is ongoing including assessments of the development and survival of outplanted corals, annual photo-quadrats to assess changes in benthic composition, fish and invertebrate surveys, as well as visitor surveys to assess the social impacts of the restoration work.

All of these coral gardening projects have involved extensive consultation with management and community stakeholders to ensure support and social licence [30]. Many have included large contributions from volunteers and community partners. For example, the Reef Restoration Foundation’s projects have benefitted from the equivalent of AUD 150,000 per year of in-kind time, labour and support for implementation, monitoring, maintenance and communication. Planned long-term support and monitoring will help assess effectiveness through time.

### Substrate stabilisation

Substrate stabilisation involves the direct physical restoration and implementation of artificial substrates over areas of loose, unconsolidated reef rubble [31]. Stabilisation activities are usually implemented as a response to vessel-groundings or storms that create fields of loose rubble that limit successful recruitment of corals [32].

Substrate stabilisation projects on the Great Barrier Reef are still in their trial phases. These include deployment of electrified steel mesh at Agincourt Reef 3, offshore of Port Douglas, and Reef Stars at Moore Reef and Green Island, offshore of Cairns. At Agincourt Reef 3, six 4.5 m^2^ sheets of 6 mm reinforcing steel mesh were installed with a low-voltage electrical current applied to three panels over loose rubble fields (Fig 3e) in 2018. However, the power supply proved to be technically challenging and was switched off in December 2019. The project at Moore Reef is a partnership between Mars Sustainable Solutions and tourism operator Reef Magic Cruises. Reef stars are hexagonal sand-coated steel structures that provide a stable base for coral fragments to grow [33]. The structures were deployed in two installations in June and October 2020, in a 20 x 30m area, which is expected to be filled with reef stars over the course of the next five years. The project involved the deployment of 148 reef stars populated with ‘fragments of opportunity’ (i.e., loose fragments already detached from parent colony) collected from nearby reefs. Twenty-nine species of corals from five families were used in the first installation, and 26 species from four families included in the second, with most of the species from the Acroporidae family (E. Fisher, pers. comm.). At Green Island, Reef Stars were also trialled as part of a collaboration between the GBRMPA, the Queensland Parks and Wildlife Service (QPWS), Mars Sustainable Solutions, Great Adventures, Big Cat Green Island Cruises, the Coral Nurture Program and Gunggandji Traditional Owners. There, a web of 165 reef stars on an area of unstable coral rubble was deployed and populated with more than 2600 coral fragments in 2020. An additional 200 coral fragments were also planted on suitable hard substrate using Coralclip® on an adjacent restoration site. This project enabled extensive engagement between Marine Park managers and other stakeholders. The Commonwealth and Queensland State government’s “Reef Joint Field Management Program” is becoming increasingly engaged in reef restoration efforts and is evaluating restoration ‘tools’ for possible incorporation into their field programs. Monitoring is occurring every 6 months and planned to continue until a least 2026, including assessments of coral cover, coral health, fish biomass and diversity, and other benthic organisms at restored and control sites.

### Coral repositioning

Coral repositioning involves moving and securing dislodged coral colonies back onto the reef. On the GBR, such activities were undertaken after Tropical Cyclone Debbie impacted the Whitsunday Islands in March 2017, dislodging many 1–3 m diameter *Porites* spp. bommies from the reef slope at Manta Ray Bay and rolling them high into the intertidal zone of the reef flat. With the help of contractors experienced in heavy machinery operations, QPWS and GBRMPA partnered to reposition the dislodged bommies back into the subtidal reef flat at very low tide (Fig 3c). The repositioning of the *Porites* bommies has delivered positive environmental and social benefits. Boat access has been restored to the beach, corals have recruited to the repositioned bommies, and some remnant coral tissue survived on most bommies [34]. Furthermore, the bommies provide three-dimensional habitat structure on the outer reef flat, supporting reef fishes and other marine life. As the coral community re-colonises the surface of the bommies, it’s expected that the shallow-water snorkelling experience for tourists will improve [34]. Further yearly monitoring of recovery on the bommies is ongoing.

### Macroalgae removal

Macroalgae removal has been trialled as an intervention to suppress its proliferation and help facilitate coral recovery in different reef regions [31, 35]. For example, in Hawaii, the combination of manual removal and herbivore addition proved to be a lasting and effective control management approach against invasive macroalgae [35]. On the GBR, trials of macroalgae removal have been concentrated on fringing reefs surrounding Magnetic Island, which have demonstrated a shift from coral to macroalgal dominance [36]. In pilot studies, QPWS and GBRMPA tested manual and airlift pump algae removal systems. Manual removal resulted in an immediate reduction of macroalgae cover from 38% to 7% [30], although long-tern benefits were not quantified. The airlift pump trials (Fig 3g) require further testing to optimise the system. Simultaneous to this pilot work, a longer-term macroalgae removal study has been implemented and ongoing since 2018 around Magnetic Island. Twenty-four permanent 25 m^2^ plots were established, baseline surveys were undertaken, and algae were removed by hand from half of the plots prior to mass spawning of corals. Removal of algae was repeated approximately every six months, with austral winter removal events targeting the algal holdfast, and spring removal events targeting bulk removal of biomass before mass coral spawning. Monitoring of plots occurred every two to three months and included algal density, canopy height, benthic community composition (using photo quadrats and a modified PIT method; [37]), coral recruitment (using settlement tiles and *in situ* surveys), fish abundance and fish community composition, turf algal height, sediment deposition, and bleaching susceptibility. Removal of algae from the treatment plots has resulted in declining algal biomass through time, suggesting that regrowth is suppressed with repeated removal events [38]. Plots that had macroalgae removed also had three times more coral recruits than control plots indicating that macroalgae removal could support coral recruitment and recovery [38].

In 2019, the macroalgae removal experiment at Magnetic Island was expanded to incorporate trials of larval-based restoration [39–41], dividing twelve 25 m^2^ plots per bay into four treatment groups (i.e. control, macroalgae removal only, larval-based restoration only, and combined macroalgae removal with larval-based restoration). Following the successful release of >2 million larvae into six plots at one bay in 2019, the 12 treatment plots across both bays received >2 million larvae in 2020. Monitoring and data analysis are ongoing, and genetic analyses of successful recruits are planned to confirm their genomic origins and assess if population genetic diversity is affected through the process of larval release.

Removal of macroalgae is an activity that is highly accessible to local communities as the technique requires minimal training. As part of the research program at Magnetic Island, a partnership with Earthwatch Institute and Mitsubishi Corporation involves citizen scientists in the removal and monitoring process. Activities involving the Wulgurukaba traditional owner group as well as the GBRF-funded Community Action Plan group are in development as of 2022.

### Larval-based restoration

Larval-based restoration (also referred to as larval restoration, larval enhancement, sexual-propagation, and larval ‘reseeding’) involves supplying large numbers of coral larvae to damaged reef areas, thereby artificially increasing larval settlement and recruitment [41]. Larvae are either reared *in situ* within floating enclosures on reefs [41] or *ex situ* in laboratories or aquaculture facilities to optimise production and retain them for restoration, rather than allowing them to be dispersed in currents away from target reef areas [42, 43]. When larvae are competent to settle, they are released onto reefs using various methods, including temporarily contained under mesh sheets or tents for small scale manipulative experiments [16, 40, 44], released as larval clouds directly onto damaged reef areas [45, 46], or by using underwater robotic vehicles to release larvae very close to reef surfaces. Larval-based restoration is applied on reefs around the world and harnesses the capacity of corals to generate millions of offspring at predictable times, reduces mortality during early life stages, and minimises loss from larval dispersal [10, 40, 43, 44, 47].

On the GBR, the first larval-based restoration trials occurred on Heron Island reef patches in 2016 with philanthropic funding support from the Great Barrier Reef Foundation. *Acropora* larvae reared *ex-situ* and settled in replicate 4 m^2^ plots, resulting in increased settlement and recruitment densities in higher larval-density treatments and no natural recruitment in control plots [48].

Subsequently, larger-scale reef trials were initiated on 100 m^2^ patch reefs at Heron Island and One Tree Island reefs in partnership with GBRMPA and QPWS using multispecies mixes of *Acropora* and Merulinidae larvae [49], and on reefs in the northern GBR in partnerships with researchers, tourism operators and Traditional Owners [46]. Combinations of larger floating spawn catchers and floating larval pools were used to collect multispecies spawn slicks (Fig 3d), and more than 100 million larvae were reared in floating larval pools, with competent larvae subsequently settled onto degraded reef areas with low coral cover [46]. Some larvae were also co-cultured with Symbiodiniaceae photosymbionts to potentially enhance energy supply during and after settlement. As with the previous smaller-scale larval-based restoration reef trials, these trials significantly increased larval settlement in larval-enhanced restoration plots compared with control plots [46]. Monitoring of longer-term recruitment and coral restoration outcomes is ongoing.

Another approach for scaling up larval-based restoration aims to harvest embryos from highly concentrated wild coral spawn slicks [47]. This process involves the transfer of the slicks into vessels with massive storage capacity such as hopper dredgers (i.e., millions of litres), and culture larvae on-board until they become competent for their unconstrained release onto large areas of denuded reef that would benefit from larval saturation (Fig 3h, [47]). It has been conceptualised to incorporate common industry practices used in oil spill remediation, dredging operations, and land-based aquaculture–thus utilising industry links needed to upscale coastal marine practices [50]. An initial feasibility study of this approach was tested in November 2018 offshore from Heron Island in the southern Great Barrier Reef [51]. The multi-disciplinary team included scientists and marine engineers in a research-industry partnership. Approximately 29 million live coral embryos were collected using an oil boom, pumped on-board a tugboat, and cultured to competency using a 50,000 L aquaculture facility over a five-day period with high survival rates observed [51]. To date, this is the largest collection of wild coral spawn slicks (average initial stocking density of 1,272 L^-1^) and cultivation to competent coral larvae in the scientific literature, and among the highest rates of survival during early development (14% survival to competency), providing evidence that further upscaling to produce billions of coral larvae for delivery to target reefs may be achievable. Other known and published studies that collected wild spawn slicks and cultured to competency include those by [16] and [52], which quantified average survival rates of 5% and ~17% from initial egg stocking densities of 5,000 L^-1^ and 840 L^-1^, using *in situ* culture ponds with total capacities of 6,000 L and 22,000 L, respectively. By culturing coral larvae in a moving vessel, billions of larvae can be transported for 1000’s of kilometres during the rearing period. During this period, other technologies such as heat hardening or introduction of beneficial coral algal symbionts to enhance early growth and survival or heat tolerance could also be incorporated. Utilising wild coral spawn slicks as an abundant source of propagules takes advantage of the species and genetic diversity available in the spawning population.

With the promising outcomes of larval-based restoration trials on the GBR, a pilot capacity building project called Boats4Corals investigated whether boats and crews from the reef tourism industry and Indigenous Traditional Owners in the Whitsundays region could undertake larval restoration operations using modified larval culture pools on reefs [41, 46], initially under guidance from researchers. This pilot study, which commenced in 2020, involved redesigning some of the equipment and technology to be safer, and more easily used by stakeholders, as well as developing robust standard operating procedures, and a training workshop and materials. So far, positive engagement of the tourism industry and Traditional Owners in this project is an encouraging result for the potential to scale-up larval-based restoration operations in future spawning events by mobilising the largest source of vessels on the GBR.

### Coral seeding

Coral seeding, defined as the settlement of coral larvae or the attachment of small fragments or micro-fragments (<1 cm^2^) onto devices for deployment, aims to assist reef recovery and restoration by enhancing the low natural survival characteristic of post-settlement and post-deployment phases (reviewed in [43]). While similar to larval-based restoration, coral seeding includes the attachment surface in the deployment, which offers several potential benefits, including: (i) a short (< 12 week) protective grow-out phase prior to deployment, either in an aquaculture facility or an *in situ* nursery, (ii) the ability to manipulate the benthic community surrounding the coral to control settlement inducers and competitors, (iii) the opportunity to overcome challenges associated with natural settlement processes on degraded reefs, including a lack of appropriate settlement cues or available substrate [53–55] and the presence of settlement inhibitors [56–58], and (iv) to incorporate design features that enhance survival during the most vulnerable sessile phase of a coral’s life-history [59–63]. Coral seeding has been developed in the Indo-Pacific using natural rubble-like substrates [64] or specifically engineered substrates [65] and 3D printed tiles [45]. More recently, SECORE International is also pioneering coral seeding work in the Caribbean and Central Pacific [61]. Building on this work, and in collaboration with SECORE and others, new research in Australia is looking to (i) optimise device design (i.e. material type, shape, density, retention, delivery method), (ii) understand device by species by environment interactions, and (iii) operationalise the technique for automation and high throughput production.

Recent research, funded by the Australian Institute of Marine Science (AIMS), has identified some functional features, such as wide grooves in the settlement surface, that improved *Acropora tenuis* spat survival three-fold over featureless devices [62, 63]. A second trial, jointly funded by AIMS and the Great Barrier Reef Foundation and undertaken on a central mid-shelf reef of the GBR, found that the environment was more influential than the device design in driving coral survival, with yield (survival at the device level) averaging around 25% across device types over 219 days, but three times higher at one site compared with the other sites [66]. Building on this work are subprojects within RRAP and the Keppel Islands Coral Project, a five-year industry funded project underway at AIMS to trial the deployment of seeding devices that optimise coral survival and growth (Fig 3i), and to understand the environmental factors that contribute to early life history bottlenecks in coral survival. Early trials in 2019 identified high rates of mortality of seeded recruits over a four-month outdoor aquarium holding period, suggesting that survival may be optimised by short holding periods and early outplanting. The relative benefit of outdoor versus indoor holdings for coral recruits of different species is also under further investigation. Aragonite plugs seeded with recruits from three coral species common in the Keppel Islands, *Acropora millepora*, *Acropora muricata* and *Montipora aequituberculata* were deployed across three sites in the inshore Keppel Islands in January 2021 (~ six week old spat). Surveys of devices in April 2021 indicated early survival yields averaging 50% on plugs and >95% on devices, and differences in survival amongst sites. Ongoing surveys of devices are planned for throughout 2021, and aim to identify the drivers of site and species-level differences in survival and growth to allow future optimisation of coral seeding restoration efforts. Taken together, these projects demonstrate the capacity to improve the survival of deployed spat and coral fragments above natural rates and suggest that seeding devices may offer an additional tool that, when combined with other techniques and advances in automation and high throughput technology, may offer a valuable tool for the reef restoration toolbox.

### The Reef Restoration and Adaptation Program

The Reef Restoration and Adaptation Program (RRAP), was initially funded by the Australian Government to scope existing and novel technologies that could help support the resilience of the GBR, and to deliver an investment case for further research and development of reef interventions [23, 67]. This feasibility study involved social scientists, coral reef ecologists, geneticists and biologists, economists, modellers, engineers, mathematicians and governance and program delivery experts. Importantly, the program was co-designed with the relevant regulator, the GBRMPA.

**Fig 4:**
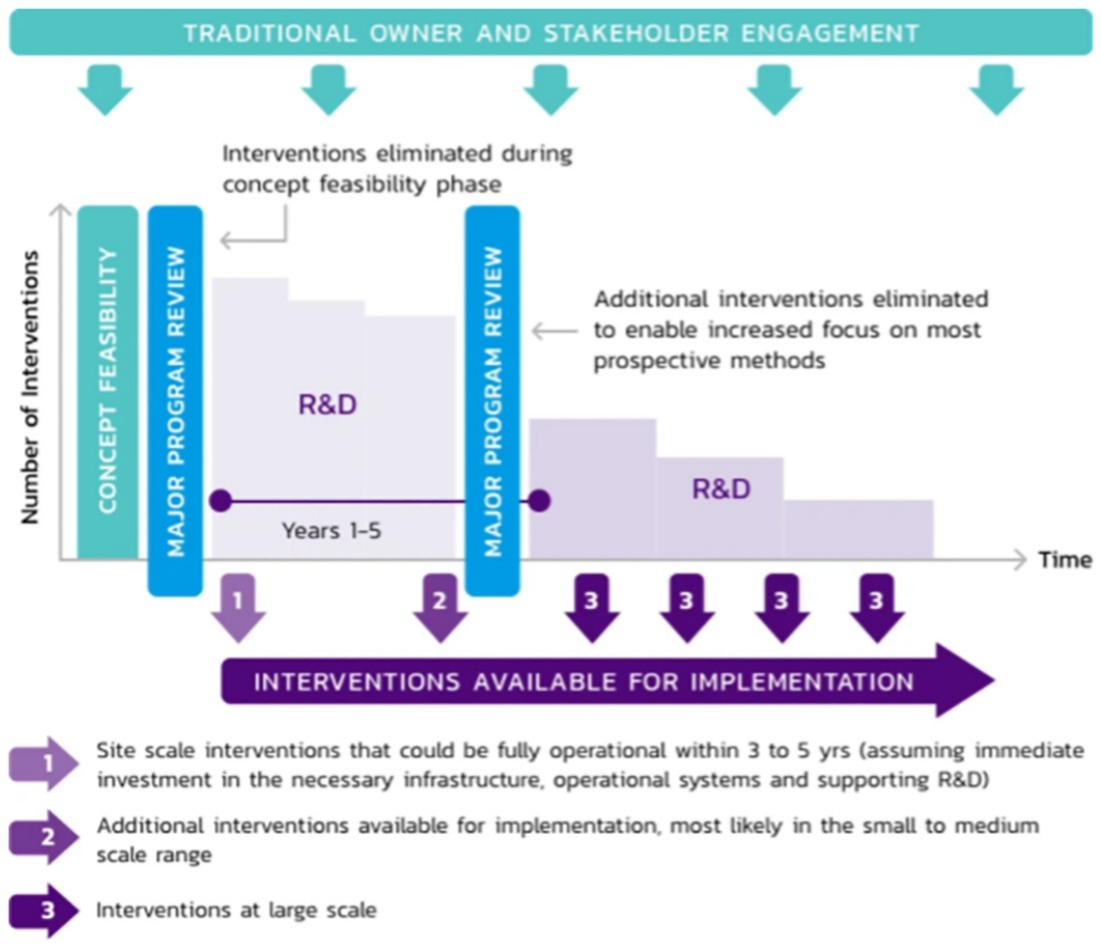
RRAP strategy to progressively deliver interventions and refine the focus of the R&D program as research findings improve knowledge of feasibility, risks, efficacy, social acceptance and regulatory compliance.

The RRAP feasibility study found there was no single solution to supporting the Reef’s resilience [67, 68]. Rather, a range of methods would be needed to work together to provide compounding benefits, along with ongoing best-practice reef management and drastic greenhouse gas emission reduction. The concept feasibility program investigated 160 possible interventions across a range of scales. Of these, 43 were recommended for further investigation. The second phase, the RRAP Research and Development Program, began in 2020 to investigate the identified prospective techniques, quickly eliminating those found not feasible, and progressively developing and field-testing the most promising over two five-year periods. This phase will also include continuous Traditional Owner, stakeholder and community engagement (Fig 4). This first research and development phase is funded through the $100M allocated for reef restoration and adaptation science as part of the $443.3M partnership between the Australian Government’s Reef Trust and the Great Barrier Reef Foundation with additional contribution from research partners.

**Fig 5:**
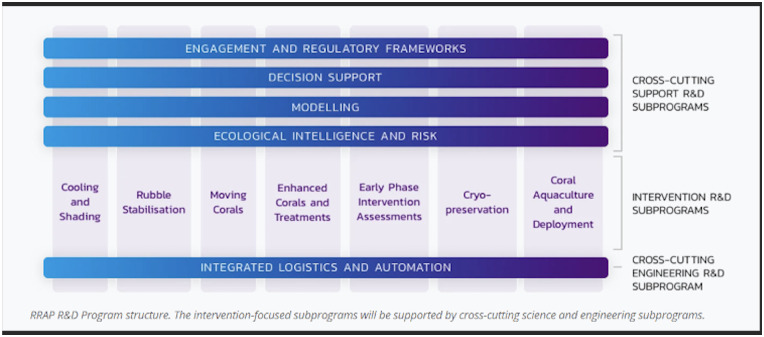
The core of the RRAP Program is founded on seven intervention research and development subprograms, including: (1) cooling and shading, (2) rubble stabilisation, (3) moving corals (larval-based restoration), (4) enhanced corals and treatments, (5) early phase intervention assessments, (6) cryopreservation, and (7) coral aquaculture and deployment. These are supported by crosscutting R&D subprograms, engagement and regulatory frameworks, decision support, modelling, and ecological intelligence and integrated logistics and automation.

The targeted research and development program will balance minimising risk with maximising the opportunity to save coral species and values to create a suite of interventions that would take an integrated, three-pronged approach: 1) cooling and shading the reef to help protect it from the impacts of climate change 2) assisting reef species to adapt to the changing environment, and 3) supporting the recovery of degraded reefs through restoration; over seven intervention research and development subprograms (Fig 5). The focus would be on the prevention of degradation and assisted adaptation to minimise the need for restoration, which was found to be expensive and more difficult to achieve at scale.

The envisaged final phase of RRAP would be an implementation phase which could overlap with the research and development phase as early as five years from commencement, depending on the specific intervention. This would include commercial transfer of the reduced set of candidate intervention measures, including the construction, operation, and deployment of full-scale systems.

## Novel interventions to enhance coral performance under climate change

The current and projected rates of climate warming combined with the devastating effects of heat-induced coral bleaching on reef health indicate that corals need to rapidly adapt to become more thermally tolerant if they are to survive into the future. Assisted evolution is a collective term for several interventions aimed at improving coral performance through the acceleration of naturally occurring evolutionary processes [69]. To date, it has mostly focussed on enhancing thermal tolerance but in principle other traits such as growth could be targeted. Other genetic management actions focus on enhancing adaptive potential, mostly through the maintenance of genetic diversity [70]. A major cost and scale driver for reef restoration and adaptation approaches is the mortality of corals in the first year of life, which can be in the order of 80–99.9% (reviewed in [43]). Thus, any intervention that can improve growth and survival of corals can greatly improve the feasibility of restoration and adaptation activities. Given that coral survival and reproductive maturity are linked to colony size and age [42, 71], growth rate is a critically important component of adaptation, particularly as summer temperature maxima (even in the absence of a heat wave) are increasing with climate change.

There are three main approaches to assisted evolution:

**Managed or selective breeding** refers to approaches based on sexual reproduction, such as cross-breeding between distinct populations of the same species (also known as intraspecific hybridisation) or between species (also known as interspecific hybridisation), and the cross-breeding of comparatively heat tolerant colonies from the same population [72]. These approaches all have the aim of selectively restoring tolerant corals onto reefs to increase the performance of the receiving population. The first and third approach could be achieved through the movement of adult colonies or adult colony fragments, as it is expected that the translocated individuals will eventually interbreed with the native populations. An increasing body of theoretical and lab-based work suggests the potential benefits of this approach [73–77] especially if the deployment strategies are optimised to local conditions [78].
**(Pre)conditioning** is the exposure of organisms to sublethal stress which may elicit an increased tolerance to subsequent stress exposures. Prior exposure to elevated temperatures can reduce bleaching susceptibility in adults (e.g., [79, 80]) and experiments in progress suggest the same can be achieved through the larval duration of some species [81]. Conditioning can occur through a variety of mechanisms, for example, frontloading of heat stress genes in the coral host due to epigenetic changes [82], or changes in microalgal symbionts (Symbiodiniaceae, e.g., [83, 84]). However, it is unclear how long the tolerance traits through preconditioning will be maintained, and if there are reductions in other fitness traits across life stages and generations.
**Microbiome manipulation or microbiome engineering** is the manipulation of individual microbes, microbial communities, or their hosts (specifically, host-based mechanisms of microbial recognition, [85]). Promising results have been obtained through the laboratory evolution of cultured Symbiodiniaceae, followed by reintroduction of heat-evolved strains into coral hosts, some of which enhanced thermal bleaching tolerance of coral larvae relative to larvae with wild-type algal symbionts [86]. The development of bacterial probiotics is another approach currently being explored [87], but this field is in its infancy [88–91].

A less often discussed assisted-evolution approach is the managed formation of coral chimeras, for instance through the use of microfragments of full siblings to reskin dead skeletons of large, massive colonies [92]. Close relatives are likely able to fuse once growing microfragments come into contact with one another [93], and each sibling will have distinct allelic combinations and trait values. This may result in the chimeric colony being more stress resistant, however, this needs to be tested experimentally.

Currently, assisted evolution for corals is in a research and development phase and is not yet implemented on the GBR. The feasibility of assisted evolution has mostly been tested under laboratory conditions and in small field trials (Fig 3b; e.g., [73, 76, 77, 86, 94–97]). Larger field trials are underway (e.g., [98]) but not yet comprehensive among coral sources and recipient reefs.

Coral populations that have survived bleaching or thrive under present day ‘naturally extreme’ reef environments are a key genetic resource with which to evaluate and potentially enhance stress tolerance for restoration efforts [99]. On both the east and west coast of Australia, coral species (including many species of *Acropora* impacted by recent heat waves) can exist along broad latitudinal gradients stretching >2000km, exposing species to >7°C temperature difference. Such broadly distributed coral species typically exhibit different thermal optima tuned to local conditions (e.g. [100]) that appear to reflect heritable differences in factors promoting thermal tolerance under local conditions such as latitude [74]. Reefs in shallow areas have thermal histories that are generally warmer and more variable (reviewed in [101]). Whilst higher average temperatures within shallow systems can enhance thermal tolerance (e.g., [102, 103]), “night time reprieves” are likely also critical in aiding overall resilience [104]. For example, under the most extreme cases, such as the 10 m tidal amplitudes of the Kimberly region of western Australia, corals can be frequently exposed to temperatures >35°C, with 7°C daily fluctuations [105]. However, even on shallow inshore reefs of the GBR, where thermal histories are warmer (e.g., by at least 2–3°C, Howells et al. 2012) and/or more variable [106], some corals appear to thrive. This is likely to be influenced by both host genetic factors, and differential associations with microbial consortia, including more thermally resistant Symbiodiniaceae [107].

Corals within shallow nearshore environments, and particularly. Those that neighbour (or penetrate in) mangrove lagoons, are exposed to variable environmental conditions including changes in pH (often more variable and/or on average less alkaline), extremes in temperature, and amplified metabolic activity [101, 108, 109]. The most extreme examples occur for shallow reef flats that neighbour (or penetrate into) mangrove lagoons where coral populations thrive within exceptionally lower pH, warm but also routinely deoxygenated environments [108], including on the GBR [109]. Corals conditioned under such extremes, and notably species of *Acropora*, have been proposed as the best models to identify traits and trade-offs required for corals to survive future climate scenarios, given that the mangrove lagoon imposes simultaneous effects of ocean warming, acidification and deoxygenation [101], yet appears to also remain resistant to heat waves (e.g. the 2016 mass bleaching event in New Caledonia, [108]). These studies can make valuable contributions to our understanding of environmental trade-offs to inform coral seeding activities to ensure that tolerance to one stressor does not come at a loss of that to another co-occurring condition (e.g., [110, 111]).

Together these various examples of naturally heat tolerant corals demonstrate existing natural variation in thermal tolerance and therefore provide important genetic reservoirs to support reef restoration and adaptation activities. However, key knowledge gaps remain. For example, the impacts of selective breeding on genetic diversity and local adaptation, or the capacity for corals to maintain traits among generations still warrant further studies. Reciprocal transplant and gardening experiments of populations and genetic crosses of corals are now underway to understand genetic and environmental drivers of coral fitness among reefs (e.g., [98]). Ultimately these efforts will need to resolve the patterns and processes of reproduction and connectivity.

## Social perspectives on coral restoration in Australia

Recent studies clearly describe how the impacts of climate change, such as major bleaching events, are keenly felt by reef users and visitors. They report diminished experience quality, less confidence that their own actions will matter, and emotional responses consistent with ‘ecological grief’ [112]. These insights point to some of the negative social implications of damaged ecosystems and also encourage the consideration of social benefits (i.e. psychological, collective and cultural) that are possible through restoring coral reef ecosystems. There have been recent calls in the restoration ecology literature for a better understanding of (and capacity to measure) the social benefits of restoration projects [113–115]. Internationally, in development contexts, coral restoration initiatives can sometimes engage with the livelihood or socio-economic dimensions of restoration. However, these are often secondary to, or a means towards, achieving ecological outcomes, or assessing the feasibility of restoration techniques [113].

Within Australia, as elsewhere, there are programs of community action and partnerships between citizens and researchers that encourage participation in monitoring of coral health and in coral restoration activities. These efforts often intersect with long-standing marine citizen-science programs that are sponsored by non-governmental organisations, education and research providers or agencies with responsibility for marine park management. This can contribute to environmental literacy and citizenship amongst participants [116]. Furthermore, it has been argued that citizen-science practice can provide community voice and influence in decisions about managing long-term ‘landscape-level’ environmental changes [117] and may contribute to a social licence for marine conservation research and intervention [118]. It has been reported, however, that translating local activity and outcomes into broader strategic management impacts for marine ecosystems can be problematic [119].

When approaching politically and socially contested proposals such as large-scale restoration and adaptation of the GBR, there are a number of complex social and institutional considerations. The first of these is understanding the social acceptability (or otherwise) of proposed restoration interventions amongst the broader community, key rights-holders such as Indigenous Traditional Owners, stakeholders such as reef-using or reef-dependent economic sectors, and other engaged interest groups. The second challenge is assessing how proposed interventions (or non-intervention) are likely to affect the diverse social and cultural values, uses and benefits for those groups that associate with the Reef. Third, there is a need to develop appropriate and meaningful ways to engage different groups and interests in the design, deployment and evaluation of proposed interventions or technologies over time. A recent review identified several distinct types of challenges researchers face when working on assisted regional ecosystem adaption including scientific conflicts and debates over the “facts”; social and governance challenges; epistemic challenges, and ontological conflicts [120]. Addressing these challenges requires appropriate engagement strategies.

There are normative reasons (i.e. societal expectations about what “should happen” or is believed “right”) and instrumental reasons (i.e. what is needed to achieve a particular goal) why understanding social acceptance, assessing likely impacts on diverse values, and designing appropriate engagement processes are important. They are normatively important because there are democratic, moral and ethical obligations to involve people in decisions and actions that may affect their livelihoods or future well-being. They are also instrumentally important in that good participation or co-design can lead to enhanced performance of the planned environmental intervention through better tailoring to local conditions, the use of local and traditional knowledge and reduced stakeholder conflict [121]. Critically, however, these place or interest-based engagement activities need to connect with, and influence, the broader public debate and governance systems (including regulatory) intended to steer large-scale restoration efforts [122, 123]. These considerations will become increasingly important for how marine researchers and their organisations operate in the near future. Research providers and investors in Australia are recognising that greater levels of communication and co-design are fundamental to meeting any expectations of a more responsible research and innovation agenda for restoration [124].

## Summary and future directions

Since 2017, there have been approximately 19 in-water coral reef restoration projects, and a rapidly growing field of research into coral restoration and adaptation on the GBR including RRAP. This reflects an increasing urgency for action to confront the coral reef crisis, and an increase in funding opportunities and management appetite for the implementation of interventions. Generally, there has been a collaborative, positive spirit, and coral restoration has brought together stakeholders who rarely work together [125]. However, many of these projects are still in their very early stages, and are likely to experience challenges as they continue to rapidly develop. As they develop in the coming years, specific goal-based monitoring, and assessments of cost-effectiveness, scalability, and socio-economic impacts will be necessary. Lessons learned and potential technology transfer will ultimately benefit the field of coral reef restoration globally.

Australia has about 50,000 km^2^ of coral reefs [126] and no existing techniques could possibly be scaled up to cover even a fraction of this area. Larger scale coral restoration and adaptation is relatively new to Australia and may offer new hopes for corals under climate change [23]. It is not, however, a silver bullet and the best futures for coral reefs include the trio of emissions and stress reduction, management of local factors (e.g., zoning and enforcement), and active interventions (e.g., coral reef restoration, predator control) [127]. Coral restoration and adaptation in Australia shows exciting potential to restore local-scale sites with existing technology and buy time while we develop more scalable tools and take urgent global action on climate change.

Additionally, there is also value in small-scale interventions, particularly at high-value sites where objectives of restoration align with economic and/or cultural incentives through increasing community engagement or the amenity value of tourism hotspots. Since 2018, at least ten tourism organisations have been actively involved in coral restoration projects on the GBR, motivated by the desire to be proactive in improving the condition of the reefs they use [128]. While the majority of these activities have always been able to be permitted, the development of interventions policy and guidelines has facilitated a pathway for more focused and evidence-based proof-of-concept research into interventions to occur. This has provided direction for appropriate stewardship activities to be trialed in different environments to determine the level of success. Consequently, scalable efforts are being delivered via growing networks collectively across sites, as opposed to building scale solely within one site.

The novelty of the many intervention strategies presented here represent an important shift in the use of coral restoration—away from assessing the feasibility and efficacy of individual techniques, towards developing a holistic approach that includes coordinated and practical solutions with specific objectives linked to long-term outcomes in a changing climate. In Australia, this shift is greatly facilitated by large investments through the RRAP Program. There has also been a shift from research and development to implementation. This approach requires research partnerships that span beyond the coral research space to include Traditional Owners, engineers, social scientists, modellers, economists, infrastructure development experts, and project managers.

The rapid progress in trialling coral restoration methods in Australia builds on decades of overseas experience. Australia is now investing considerable time and money into developing novel interventions and consequently, is fast becoming an emerging leader in this space. There are great advantages to continuing to actively collaborate with researchers internationally and with other sectors not currently involved in coral reef management. This could be facilitated through coordinating organisations and networks such as the Coral Restoration Consortium, the International Coral Reef Initiative, RRAP, the United Nations, and the International Coral Reef Society to hasten the optimisation of reef restoration interventions and their contribution to future increases in reef resilience.

The challenge for the future include: (1) demonstrating that active restoration can play a meaningful role in improving reef condition through supporting resilience and increasing adaptation when other, less interventionist, management strategies have not worked, (2) implementing these interventions at larger scales, and (3) understanding and mitigating any potential risks from active restoration. At any scale, coral restoration and adaptation interventions do not take away the need for urgent reductions in greenhouse gas emissions.

## Supporting information

S1 Appendix (DOCX)
