# Supplementary material for: Coral restoration and adaptation in Australia: The first five years
Source: PLoS One. 2022 Nov 30;17(11):e0273325. doi: 10.1371/journal.pone.0273325 (PMC9710771; doi:10.1371/journal.pone.0273325)
Supplement: S1 Appendix — (DOCX) [file pone.0273325.s001.docx]

Coral Restoration and Adaptation in Australia

Appendix 1: List of in-water coral reef restoration projects on the Great Barrier Reef Australia as of January 2022. “/” indicates collaboration among different organisations

| **Project Lead** | **Type** | **Location** | **Latitude** | **Longitude** | **Region** |
| --- | --- | --- | --- | --- | --- |
|  |  |  |  |  |  |
| **SCU** | Larval enhancement - floating pools, culture tanks | Heron Island | -23.442298 | 151.9148 | South |
|  |  |  |  |  |  |
| **CSIRO/RRAP** | Larval enhancement - vessel | offshore Heron Island | -23.401971 | 151.939257 | South |
|  |  |  |  |  |  |
| **SCU** | Larval enhancement - floating pools, culture tanks | Vlasoff Cay | -16.657316 | 145.990598 | North |
|  |  |  |  |  |  |
| **SCU** | Larval enhancement - floating pools, culture tanks | One Tree Island | -23.507335 | 152.091509 | Central |
|  |  |  |  |  |  |
| **JCU/Reef Ecologic/SCU** | Macroalgae removal, larval enhancement | Magnetic island | -19.135865 | 146.842356 | Central |
|  |  |  |  |  |  |
| **RRF** | Coral gardening | Fitzroy Island | -16.933667 | 145.994347 | North |
|  |  |  |  |  |  |
| **Reef Ecologic** | Coral gardening | Blue Pearl Bay | -20.04814782 | 148.8803971 | Central |
|  |  |  |  |  |  |
| **Reef Ecologic** | Coral gardening | Manta Ray Bay | -20.0607372 | 148.9570286 | Central |
|  |  |  |  |  |  |
| **QPWS** | Coral repositioning | Manta Ray Bay | -20.0607372 | 148.9570286 | Central |
|  |  |  |  |  |  |
| **Reef Ecologic/Quicksilver** | Substrate stabilisation - Electrochemical | Agincourt reef 3 | -16.05 | 145.833333 | North |
|  |  |  |  |  |  |
| **UTS/Wavelength/CNP** | Substrate stabilisation and coral gardening | Moore Reef | -16.88763088 | 146.1903802 | North |
|  |  |  |  |  |  |
| **Mars/Reef Magic** | Substrate stabilisation- Reef Stars | Moore Reef | -16.88763088 | 146.1903802 | North |
|  |  |  |  |  |  |
| **GBRMPA/QPWS/Mars/GreatAdventures/Big Cat/CNP/Gunggandji** | Substrate stabilisation - Reef stars, Coral Clip | Green Island | -16.76202146 | 145.9718207 | North |
|  |  |  |  |  |  |
| **UTS/Wavelength/CNP** | Coral gardening - Coral clip | Opal Reef | -16.210456 | 145.881327 | North |
|  |  |  |  |  |  |
| **UTS/Wavelength/CNP** | Coral gardening - Coral clip | Hastings Reef | -16.51732586 | 146.014384 | North |
|  |  |  |  |  |  |
| **UTS/Wavelength/CNP** | Coral gardening - Coral clip | Low Isles | -16.38647142 | 145.5633709 | North |
|  |  |  |  |  |  |
| **UTS/Wavelength/CNP** | Coral gardening - Coral clip | Mackay Reef | -16.04431834 | 145.6481283 | North |
|  |  |  |  |  |  |
| **UTS/Wavelength/CNP** | Coral gardening - Coral clip | Upolu Reef | -16.67584484 | 145.9407822 | North |
|  |  |  |  |  |  |
| **RRAP** | Larval seeding | Keppel island | -23.17483192 | 150.9584145 | South |
